# The treatment of melasma by silymarin cream

**DOI:** 10.1186/1471-5945-12-18

**Published:** 2012-10-02

**Authors:** Tagreed Altaei

**Affiliations:** 1 Department of Pharmacology and Toxicology, College of Pharmacy, Hawler Medical University, Erbil City, Kurdistan, Iraq

**Keywords:** Silymarin, Melasma, Sunlight

## Abstract

**Background:**

Melasma is an acquired increased pigmentation of the skin characterized by symmetrical and confluent grey-brown patches usually on the areas of the face exposed to the sun. Silymarin strongly prevents photocarcinogenesis, and significantly prevented melanin production. The objectives of this study were the assessment of safety and efficacy of topical Silymain (SM) cream in a double-blind placebo controlled study for treatment of melasma patients.

**Methods:**

Experimentally on 24 Albino rabbits were randomly divided into 4 equal groups. [A] No treatment, [B] received placebo, [C] treated with SM cream (0.1), & [D] treated by SM (0.2), were applied topically before UV sun light exposure for 30 days, assessed clinically & tissue pathology. Clinically on 96 adults diagnosed with melasma randomized to three equal groups to receive one of the tested drugs applied twice daily for 4 weeks, evaluated by the response; lesion size, melasma area and severity index score, Physician global assessment, and subjective assessment.

**Results:**

The Clinical and histopathology observations were reduced significantly in SM groups. Clinically; all patients showed significant excellent pigment improvement & lesion size reduction with SM treatments from the 1^st^ week. All patients were fully satisfied 100%. No side effects were observed.

**Conclusions:**

Silymarin showed tremendous improvement of melasma in a dose-dependent manner, and was effective in prevention of skin damage caused by U.V. sunlight. It is a safe new candidate effective treatment for melasma.

**Trial registration:**

Australian New Zealand Clinical Trials Registry - ACTRN12612000602820

## Background

Melasma is a common acquired pigmentary disorder that occurs usually in women (more than 90% of cases) of all racial and ethnic groups [1]. Melasma presents as brown to grey macules and patches, with serrated, irregular, and geographic borders [2]. The pigmented patches are usually sharply demarcated [3] and symmetrical. Melasma has a predilection for sun-exposed areas. The etiology is not entirely elucidated; however, the ultraviolet sunlight exposure appears to be the most significant factor [4]. In those patients with epidermal type melasma, there are several treatments available. Topical agents include phenols, e.g., hydroquinone; retinoids, e.g., tretinoin; azelaic acid; kojic acid; and glycolic acid [1].

Silymarin, derived from the milk thistle plant *Silybum marianum* (L.) Gaertn] is a natural polyphenolic flavonoid. Its main component silybin (silibinin), is considered to be the most biologically active with potent antioxidant properties [5]. Cutaneous photoprotection mechanisms triggered by silymarin and silybin are numerous and mainly demonstrate mainly their ability to reduce and suppress harmful effects of solar UV radiation, such as UV-induced oxidative stress, inflammation, immune responses and DNA damage as well as induction of apoptosis [6]. Silymarin significantly prevented melanin production in a dose-dependent manner with an IC50 value (concentration producing 50% maximal inhibition) of 28.2 μg/ml, without effects on cell viability [7]. Even in high doses, silymarin does not show any toxic effects and, in fact has no harmful effects on the embryo [8-10].

## Methods

### Formulation of tested drugs

Silymarin (SM), App-Chem-Bio; China prepared in cream base of different concentrations [11]. Placebo is a cream base free from active constituent.

### Experimental study

Twenty-four healthy New Zealand rabbits Albino rabbits (weight: 1500 ± 500 g each), were individually housed in suspended cages, for 1 week before the experiment. The ethics committee, before the experiment, has approved the experimental protocol. They were kept in the same environmental and nutritional conditions (temperature 25 ± 2°C, relative humidity 40%-60%, and 12 hours in light and 12 hours in darkness cycles) in the animal house of the college of medicine. At the beginning of the study, animals were randomly divided into 4 equal groups (*n*=6); group [A] did not receive any treatment, [B] received placebo, [C] treated with SM (0.1 mg/ml.kg^-1^) cream, and [D] treated by SM cream (0.2 mg/ml.kg^-1^). 3 cm^2^ of Albino rabbits' back were shaved. Then after 48 hours, the tested drugs were applied topically by cotton pad stick on the shaved area of all groups daily, 30 minutes before each UV sun light exposure. The rabbits were exposed to UV sunlight (11^+^ extreme) for 3 hours during each day of June (temperature 43 ± 2°C) for 30 days. All animals were painlessly killed by chloroform and samples were taken from the shaved area for tissue pathology study, the samples were put in formalin buffer 10%.

### Histopathological examination

All samples were cut into small blocks. These blocks of tissues were then routinely processed. These paraffin blocks were sectioned into 5 μm thick and stained with hematoxylin & eosin. The stained tissues were examined for histopathological alterations. The histopathology departments' staffs were blinded to the tested drugs.

### Clinical study

This is a double blind, randomized clinical study on patients with melasma attending the outpatient clinic of the Dermatology department of Medical City Hospital. The protocol was reviewed and approved by the ethic committee (University of Baghdad, College of Dentistry), and each participant signed a written informed consent. The inclusion criteria were adults with melasma without any topical, systemic, laser, and surgical treatment on face during the previous months. While the exclusion criteria were pregnant and nursing women, patients with history of hypersensitivity to some of the components of the formulas of the study, and coexistence of associate diseases and other pigmentation diseases, and concomitant use of other skin care products or systemic treatments. A history was taken from each patient, regarding age, gender, occupation, time of onset, history of pregnancy, contraceptive pills, and sun exposure. Patients were randomized in a double-blind manner to receive one treatment of the tested drugs; Group I (G I) SM (7 mg/ml) cream, Group II (G II) SM (14 mg/ml) cream, or Group III (G III) placebo, applied topically to the affected areas, twice daily for 4 weeks, also advised to avoid sun exposure and to use topical sunscreen with sun protection factor (SPF) of 15^+^ during the entire period of treatment and thereafter. The patients were seen regularly every week for one month for assessment; the response to treatment was rated by the size of lesions. Skin pigment evaluation by melasma area and severity index (MASI), physician global assessment (PGA); assessment of overall treatment of disease activity, used a scale from 0 to 10, by an independent observer blinded to the treated groups, and record the presence of any side effect. The Subjective assessment depending on recording improvement in patient satisfaction measures during the time course, and graded as follows: Grade 0 =not satisfied, Grade 1 =moderately or partially satisfied, Grade 2 =greatly but not fully satisfied, Grade 3 =fully or completely satisfied.

### Statistical analysis

In order to analyze the data, Chi-square test, Student *t*-test and *X*2 were used, *P* value of less than 0.05 was considered significant, data showed as mean ± SD (SPSS 18).

## Results

### Experimental study results

Some clinical features such as skin scaling, skin irregularity, erythema, skin hyperpigmentation, and edema were evaluated. The clinical observations are as follows:

*Group A* (G A), without treatments, dermal scaling, skin irregularity, erythema & hyperpigmentation, and edema were observed at the highest level (100%).

*Group B* (G B); received placebo*,* dermal scaling, skin irregularity, erythema & hyperpigmentation, and edema were observed at the level (90%)*.*

*Group C* (G C); treated with SM (0.1) cream, no clinical features were observed.

*Group D* (G D); treated with SM (0.2) cream, no clinical features were observed.

**Figure 1:**
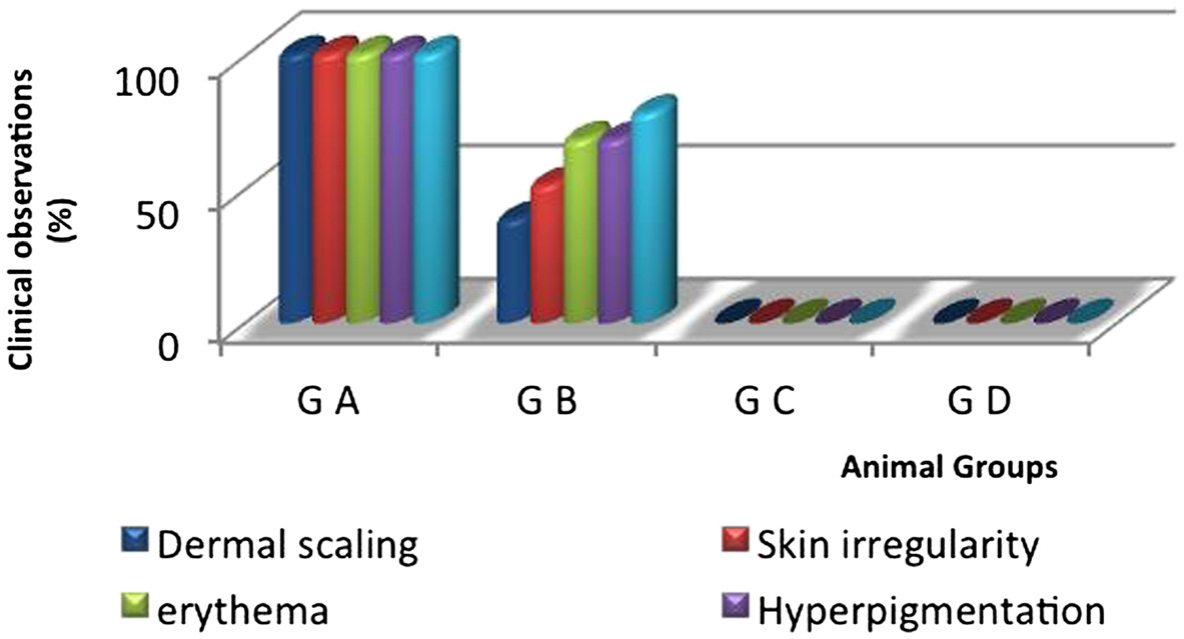
Percentage of clinical observations for animal groups after exposure to UV-sun light, level of significance [G A vs. B, C, & D groups; p=0.0001, G B vs. C, & D groups; p=0.0003, and G C vs. D; p>0.05].

The significance between groups was; G A *vs.* B, C, & D groups; p=0.0001, G B *vs.* C, & D groups; p=0.0003, and G C *vs.* D; p>0.05, as showed in (Figure 1).

### Histopathology results

**Figure 2:**
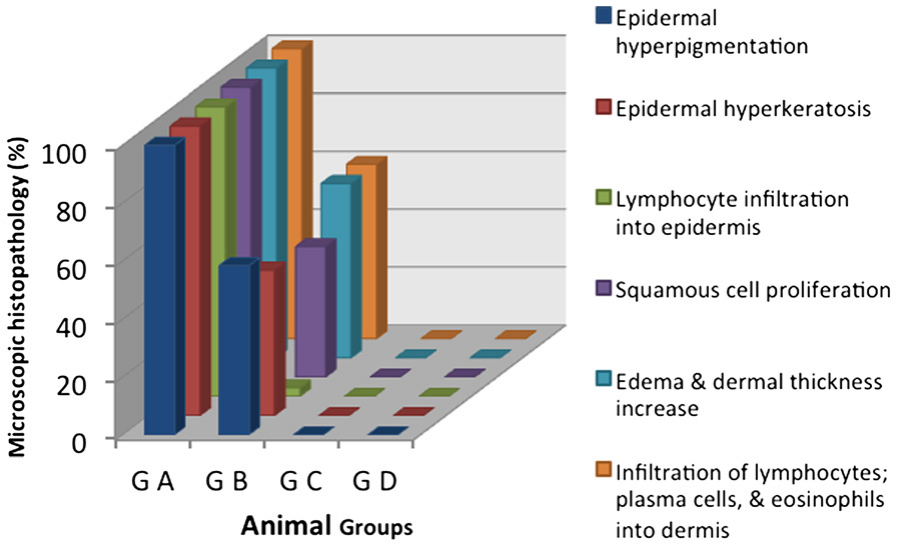
**Histopathological observations in animals' skin after exposure to UV-sun light, level of significance [G A vs. B, C, & D groups; p=0.0002, G B vs. C, & D groups; p=0.0001, and G C vs. D; p>0.05].** Group A [G A] did not receive any treatment, group B [G B] received placebo, group C [G C] treated with SM cream (0.1 mg/ml.kg-1), & group D [G D] treated by SM (0.2 mg/ml.kg-1).

Epidermal hyperpigmentation, epidermal hyperkeratosis, lymphocyte infiltration into epidermis, squamous cell proliferation, edema and dermal thickness increase, infiltration of lymphocytes; plasma cells, and eosinophils into dermis were noticed in all cases of G A (100%), in G B were (58%, 50.2%, 3%, 45%, 60.2%, 60.2%) respectively. While in G C, & G D all the microscopic observations were not seen. The level of significance was; G A *vs.* B, C, & D groups; p=0.0002, G B *vs.* C, & D groups; p=0.0001, and G C *vs.* D; p>0.05, as showed in (Figure 2).

### Clinical study results

Ninety-six melasma patients were enrolled in this study, female {F} 80 (83.3%) and male {M} 16 (16.66%), the ratio of sex distribution {F: M} within the groups were [G I (26:6), G II (27:5), and G III (27:5)]. The patients' age ranged from 28 to 55 years (median, 41 years). The duration of melasma varied from 2 to 6 years (median 4 years). Family history of melasma was found in 46 (47.916%) patients. The most frequent precipitating factors were the sun exposure (90%), and pregnancy (10%).

**Figure 3:**
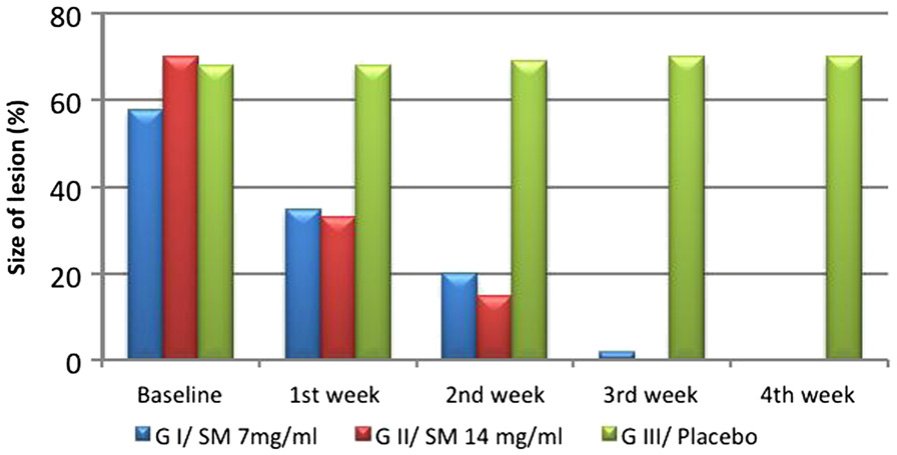
**The response size of lesion (%) at baseline and after treatment with tested drugs in melasma patients, level of significance (p=0.002).** G=group.

The response to treatment was evaluated as per the size of the lesion at the end of each week till 4 weeks. G I & G II showed significant reduction in size of the lesion at the end of 1^st^ week compared to G III, complete clearing of the lesion was shown in G II after 3 weeks of treatment, while G I in the fourth week, (Figure 3).

The average MASI score of G I before treatment was 17.1± 3.12. After treatment it changed into 12.3 ± 2.3, 6.6 ± 2.4, 0.2 ± 1.72, & 0 at the end of 1st, 2^nd^, 3^rd^, & 4^th^ week respectively. For G II before treatment was 16.5 ± 2.8. After treatment it changed into 10.4 ± 1.2, 3.5 ± 1.4, 0, & 0 at the end of 1st, 2^nd^, 3^rd^, & 4^th^ weeks respectively, and was statistically significant (P =0.0001). In G III there were no significant changes, as showed in Table 1.

**Figure 4:**
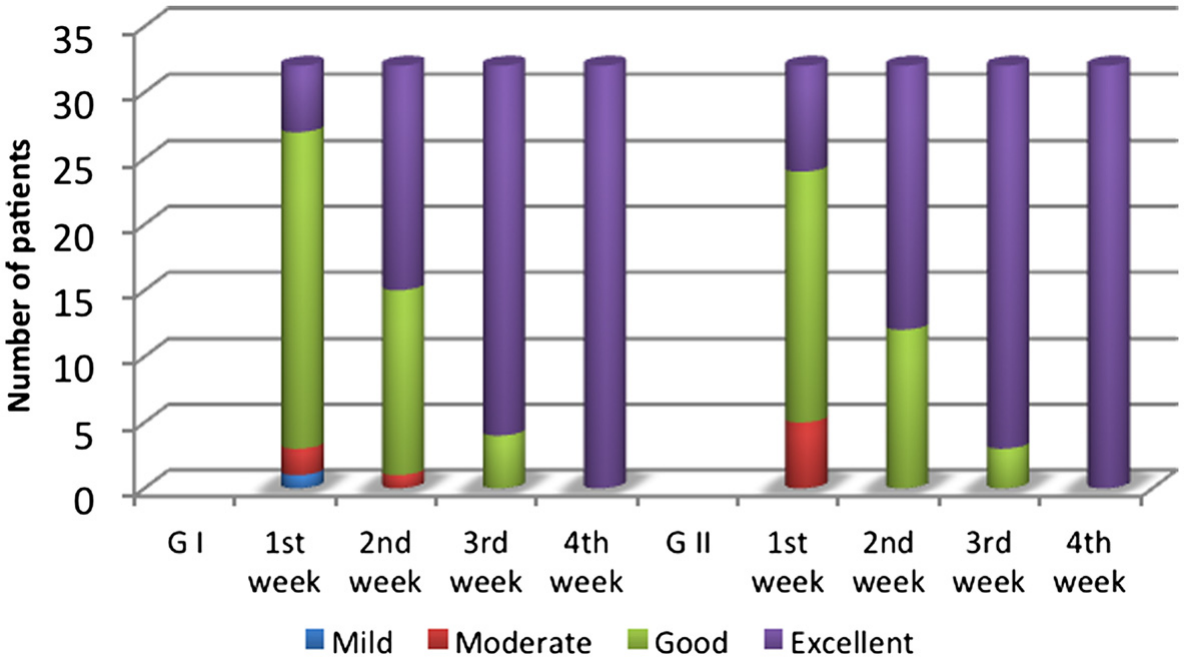
**Physicians Global Assessment (PGA) of G I (SM 7 mg/ml) & G II (SM 14 mg/ml) in treated melasma patients at the end of each week of study, (p=0.002).** G=group.

The PGA rated the G I improvement as excellent in 5 patients, good in 21, moderate in 2, and mild in 1 at the end of 1^st^ week, excellent in 17 patients, good in 14, & moderate in 1at the end of 2^nd^ week, while at the end of 3^rd^ week; excellent in 28, good in 4 patients. While the G II improvement was excellent in 8 patients, good in 19, moderate in 5 at the end of 1^st^ week, excellent in 20 patients, good in 12 at the end of 2^nd^ week; and at the end of 3^rd^ week were excellent in 29, good in 3 patients. Data showed statistical significance for SM treatments compared to placebo, (*P =* 0*.*002), (Figure 4).

**Figure 5:**
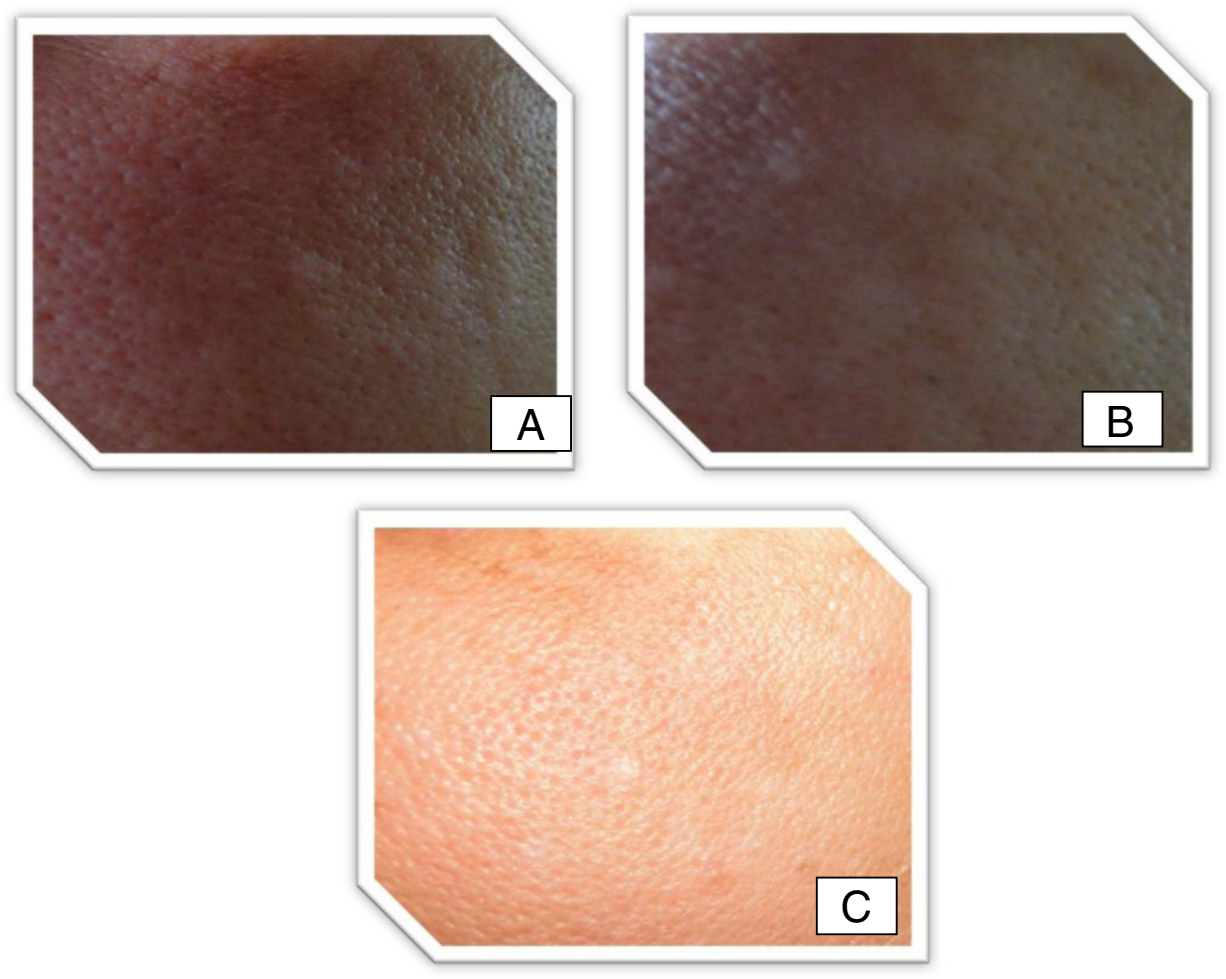
**Melasma patient (left side) treated with Silymarin.** View at onset (**A**), five days later (**B**) with an excellent improvement, and ten days later (**C**) with disappearance of melasma pigment.

Patients' satisfaction was recorded as 100% were highly & completely satisfied (Figure 5). During the period of treatment, no local or systemic adverse effects were recorded.

**Table 1:** Changes in MASI scores of tested drugs in melasma patients

		**MASI**			
**Treatment**	**Baseline**	**After 1**^st^**week**	**After 2**^nd^**week**	**After 3**^rd^**week**	**After 4**^th^**week**
G I: SM 7 mg/ml	17.1 ± 3.12	12.3 ± 2.3^a^	6.6 ± 2.4^b^	0.2 ± 1.72^b^	0^b^
G II: SM 14 mg/ml	16.5 ± 2.8	10.4 ± 1.2^a^	3.5 ± 1.4^b^	0^b^	0^b^
G III: Placebo	16.8 ± 3.2	16.8 ± 3.3^c^	16.8 ± 3.5^c^	17 ± 3.4^c^	17 ± 3.4^c^

^a^ p ≤ 0.01, ^b^ p ≤ 0.0001, ^c^ p ≥ 0.05.

## Discussion

Skin exposure to solar UV radiation induces a number of skin disorders, including erythema, edema, sunburn cell formation, hyperplasia, immune suppression, DNA damage, photoaging, melanogenesis and skin cancers. It is well documented that UV irradiation, both its UVB (290–320 nm) and UVA (320–400 nm) component, induces the generation of reactive oxygen species (ROS), which create the oxidative stress in skin cells and play an important role in the initiation, promotion and progression of skin aging and carcinogenesis. Thus, the use of antioxidants, namely naturally occurring herbal compounds, is receiving considerable interest to protect skin from adverse biological effects of solar UV radiation [6]. In the SKH-1 hairless mice silymarin inhibited UVB induced skin edema, formation of sunburn and apoptotic cells, prevented UVB-induced infiltration of inflammatory leukocytes, and significantly reduced the activity of myeloperoxidase, a marker of tissue infiltration [12,13].

Cellular antioxidant status plays a crucial role in modulating the effects of unrepaired DNA lesions and cellular sensitivity to the DNA damaging effects of solar UV radiation [14]. Benefits have been observed from both systemic and topical administration. Significant inhibition of UVB-induced sunburn, apoptotic cell formation, and edema has been associated with topical application of silymarin [12]. The present study agrees with the previous studies that the experimental study showed no clinical or histopathological features in Silymarin treated groups (both concentrations) compared to placebo and none treated groups after exposure to UV sunlight. Also, it may be one of the mechanisms of action of Silymarin in the treatment of pigmented lesion.

The body possesses endogenous defense mechanisms, such as antioxidative enzymes (superoxide dismutase, catalase, glutathione peroxidase) and nonenzymatic antioxidative molecules (vitamin E, vitamin C, glutathione, ubiquinone), protecting it from free radicals by reducing and neutralizing them [15]. Some can be inhibited by ultraviolet (UV) light [16]. It has been reported that UVA decreases intracellular glutathione status and subsequently increases UVA sensitivity of keratinocytes. The DNA-damaging effect of UVA can be reduced by improving the regulation of intracellular antioxidant status by suitable antioxidants [17].

Silymarin shows strong free radical-scavenging activity that is severalfold greater than that of vitamin E [18]. It inhibits lipid peroxidation and provides significant protection against UVB-induced depletion of catalase activity. Therefore, silymarin can effectively terminate the harmful biochemical reactions by scavenging free radicals and ROS, and by strengthening the cellular antioxidant status [19]. This may be the other mechanism of action of Silymarin in the treatment of melasma.

Silymarin inhibited l-DOPA oxidation activity of tyrosinase, the rate-limiting melanogenic enzyme, in cell based-systems, but it did not directly affect cell-free tyrosinase activity. Furthermore, Western blot analysis indicated that silymarin decreased the expression of tyrosinase protein [7]. This explains the exact main mechanism of action of silymarin in the treatment of melasma.

This study showed a significant reduction of pigment, melasma lesion in a short period of time. Silymarin has the efficacy to treat melasma in a dose dependent manner. It is safe. No side effect was observed. All patients were fully and completely satisfied from the first week of treatment with Silymarin.

The author suggests studying other pharmaceutical preparations of Silymarin like gel or paint and other dosing intervals in the treatment of melasma.

## Competing interests

The author declares that there are no competing interests.

## Authors’ contributions

TA, designed and made the whole work of this original study. The author read and approved the final manuscript.

## Pre-publication history

The pre-publication history for this paper can be accessed here:

http://www.biomedcentral.com/1471-5945/12/18/prepub
